# Recurrent malignant solitary fibrous tumor of pelvis: A case report and treatment approach

**DOI:** 10.1097/MD.0000000000034520

**Published:** 2023-08-04

**Authors:** Panpan Zhou, Xiaopei Xu

**Affiliations:** a Department of Radiology, The Second Affiliated Hospital, Zhejiang University School of Medicine, Hangzhou, China.

**Keywords:** computed tomography, magnetic resonance imaging, pelvis, recurrence, solitary fibrous tumor, treatment

## Abstract

Solitary fibrous tumors (SFTs) are rare mesenchymal neoplasms that typically arise from the pleura but can also occur in various locations throughout the body. Although there are increasingly more reports of extra-pleural SFTs, reports of SFTs in bone are very rare, and a malignant SFT accompanying recurrence of pelvis has not yet been reported.

**Patient concerns:**

We herein present the case of a 15-year-old male who experienced intermittent numbness in his right buttock, accompanied by radiating pain in his lower limbs for 6 months. Radiological examinations revealed an expansive, heterogeneous enhanced mass in the sacral and iliac regions, with a branch of the right internal iliac artery feeding the tumor.

**Diagnoses:**

The histological examination suggest a diagnosis of a malignant SFT with high proliferation activity.

**Interventions:**

The sacral mass was surgically excised.

**Outcomes:**

Following the surgery, the patient experienced a local recurrence of the tumor at 9 months and was administered adjuvant imatinib treatment. Recent magnetic resonance imaging contrast-enhanced displayed shrinkage of the tumor, which may provide certain evidence for chemotherapy for the treatment of recurrence of malignant SFTs in the pelvic region.

**Lessons:**

Complete surgical excision is the recommended treatment for this rare disease entity, and the role of adjuvant therapies is controversial due to their rarity. Our case underscores the challenges in managing recurrent malignant SFTs and highlights the importance of a thorough diagnostic workup. Further research is needed to establish the role of adjuvant therapies in the management of these tumors.

## 1. Introduction

Solitary fibrous tumor (SFT) is a rare soft tissue neoplasm that typically originates in the pleura, accounting for about 2% of all soft tissue neoplasms. However, SFTs can develop in a wide range of sites,^[1–4]^ including the head and neck, lungs, liver, and pelvis. The pelvis is a little-seen site for extrathoracic SFTs, making up 6% of all SFTs. The majority of SFTs are benign, and patients typically present with localized symptoms related to the mass effect of the tumor. Benign SFTs are usually slow-growing and have a low risk of recurrence after complete surgical resection. Malignant transformation of SFTs is relatively rare, but when it occurs, it can be associated with poor prognosis. The exact prognosis for malignant SFTs is difficult to predict, but it has been reported that the median survival ranges from 30.6 to 124.9 months with an overall 5-year survival rate of 55%.^[5]^ The rarity of malignant SFTs and their variable clinical presentation make the management of these tumors challenging.

Imaging assessment plays a crucial role in the diagnosis and management of SFTs. Computed tomography (CT) and magnetic resonance imaging (MRI) are commonly used to evaluate the location, size, and extent of the tumor. However, radiographic characteristics of SFTs are nonspecific since these tumors can display necrosis, hemorrhage, and cystic degeneration, which can mimic other malignant tumors. CT typically shows a well-defined mass with heterogeneous enhancement, whereas MRI often shows a heterogeneous mass with low to intermediate signal intensity on T1-weighted images and high signal intensity on T2-weighted images. Benign SFTs often show a pattern of slow and progressive growth, and their imaging features can be variable depending on the site of the tumor. Nevertheless, imaging assessment is an important tool for the diagnosis and management of SFTs.

Primary SFT of the bone is extremely rare, particularly in the pelvis, with only sporadic cases documented.^[6]^ This rarity makes diagnosis and management of these tumors challenging. Moreover, reports on malignant SFTs arising from the pelvis and their recurrence or response to chemotherapy are extremely rare. To the best of our knowledge, this is the first reported case of a massive malignant SFT of the pelvis. In this report, we describe the clinical presentation, radiological features, histopathological findings, and management of this rare tumor, which may provide a referential value for the treatment of similar cases.

## 2. Case presentation

### 2.1. Clinical history

A 15-year-old male teenager presented with intermittent numbness in the right buttock accompanied by radiating pain in the lower limbs for half a year. Physical examination revealed a palpable right lumbosacral mass with hard texture and poor flexion, measuring approximately 80 mm in diameter. A significant increase in bone metabolism indices (serum osteocalcin = 58.8 μg/L, serum β-collagen degradation products = 892.8 μg/L, and serum amino-terminal propeptide of type I collagen = 261.5 μg/L) was observed, while serum Fe level (9.2 μmol/L) was decreased. Serum 25-hydroxyvitamin D, erythrocyte sedimentation rate, and the full set of tumor biomarkers including carcinoembryonic antigen, alpha-fetoprotein, carbohydrate antigen (CA) 19-9, CA125, CA242, CA211, neuron-specific enolase, squamous cell carcinoma-related antigen, and prostate-specific antigen were within normal ranges.

## 3. Radiological findings

**Figure 1.**
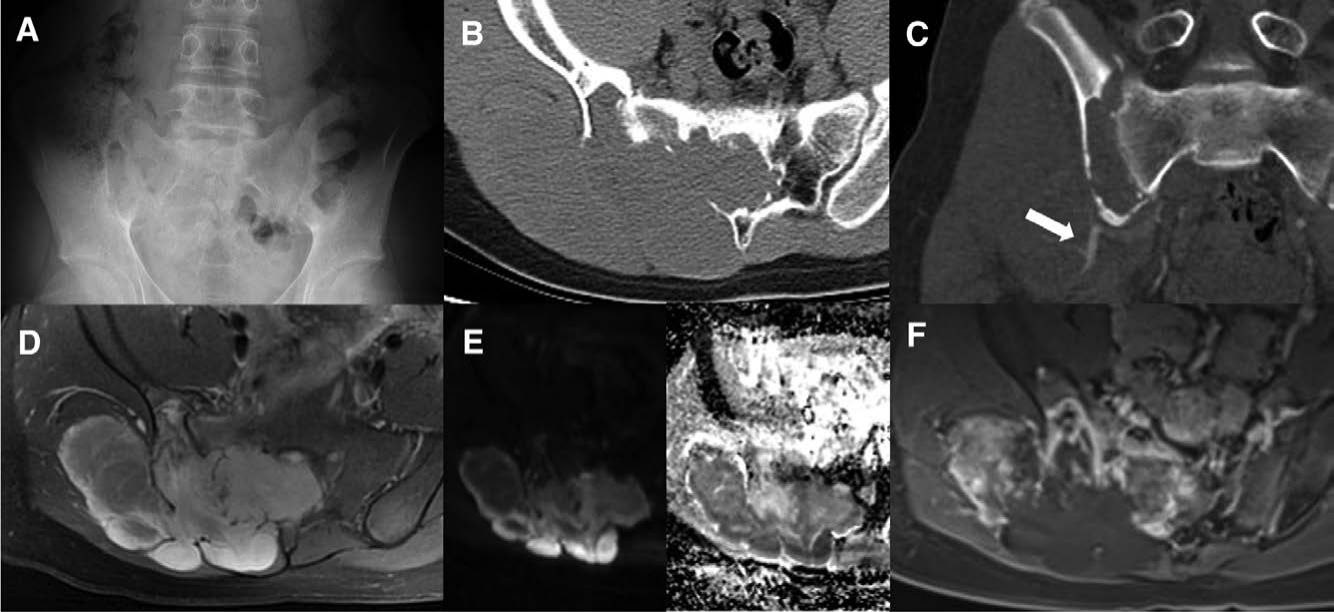
Preoperative X-ray, CT, and MRI of the SFT. (A) X-ray of lumbosacral vertebrae (anteroposterior view) manifested an expansive destruction of bone from S1 to S3 with ill-defined border, and the right sacroiliac joint space disappeared. (B) Contrast-enhanced abdominal CT scan showed a heterogeneously enhanced expansive tumor with the size of 82 × 77 × 115 mm occupying most of the pelvis with the expansion of sacral cannel and foramen. (C) Multiplanar reconstruction demonstrated that the blood supply was supplied by a branch of the right internal iliac artery (white arrow). (D) MRI exhibited a large tumor invading surrounding soft tissues, which demonstrated mixed signal on T2-weighted images with high signal dominated. (E) Diffusion weighted imaging and ADC maps demonstrated restricted diffusion with a reduction in ADC (ADC = 0.875 × 10–3 mm^2^/s). (F) Contrast-enhanced MR manifested obvious heterogeneous contrast enhancement. ADC = apparent diffusion coefficient, CT = computed tomography, MRI = magnetic resonance imaging, SFT = solitary fibrous tumors.

X-ray examination of the lumbosacral vertebrae revealed an expansive destruction of bone from S1 to S3 with an ill-defined border (Fig. 1A). Unenhanced abdominal CT scan showed an 8.2 × 7.7 × 11.5 cm expansive tumor occupying most of the pelvis with the expansion of sacral canal and foramen. Contrast-enhanced CT revealed a heterogeneously enhanced mass with evident brim enhancement and weak central enhancement. In addition, multiplanar reformation showed that the mass was supplied by a branch of the right internal iliac artery (Fig. 1B and C). Subsequent MRI exhibited a large tumor invading surrounding soft tissues, which demonstrated low intensity on T1-weighted images and mixed intensity on T2-weighted images with high intensity dominated. The mass demonstrated restricted diffusion with a reduction in apparent diffusion coefficient and MR contrast-enhanced manifested obvious heterogeneous contrast enhancement (Fig. 1D–F). The lesion was suggestive of a malignant tumor based on these imaging findings, and a surgery was scheduled. The preoperative CT diagnosis was schwannoma, while the MRI diagnosis before surgery was giant cell tumor of bone.

## 4. Surgery and pathological examination results

**Figure 2.**
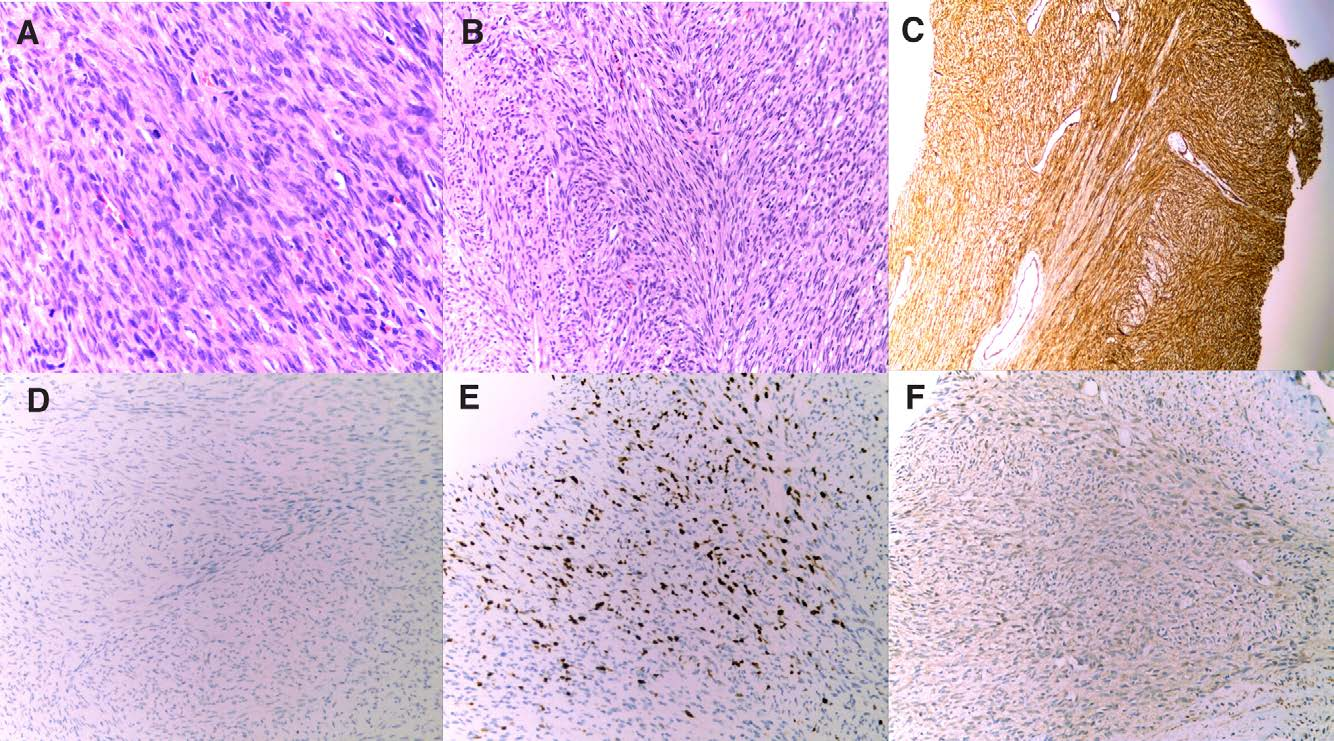
Hematoxylin and eosin-stained and immunohistochemical photomicrographs of the tumor. (A and B) Bland, ovoid to spindle nuclei cells with hyaline collagen deposition. (C) Immunohistochemical analysis for CD34. (D) Immunohistochemical analysis showed Ki67 (+, 15%). (E and F) Immunohistochemical staining was positive for S-100 and STAT6, respectively.

CT-guided biopsy revealed spindle cell proliferation with mild to moderate cellular atypia and active mitotic activity (7 mitoses per 10 high power fields). Immunochemistry was positive for CD34, CD31, CD163, SMA, caldesmon and STAT6 and negative for CD99, CK(AE1/AE3), S-100p, SOX10, WT1 and GFAP. The Ki-67 labeling index was 15% (Fig. 2). The patient underwent preoperative arteriography and embolization of the bilateral lumbar and internal iliac arteries due to the risk of bleeding during resection. The patient then underwent extensive surgical resection of the sacral tumor, followed by pelvic reconstruction, acral canal decompression, lumbopelvic fixation, and fascioplasty. A resection was performed that revealed a spindle cell tumor composed of hemangiopericomatous structures and collagenous areas. This case was initially diagnosed as a malignant isolated fibrous tumor or malignant SFT based on a combination of biopsy immunohistochemistry, tumor site, and morphology. The tumor measured 13 × 9 × 6 cm and involved the pelvic bone and soft tissues, while the margins of the bone and surrounding soft tissues were negative. Molecular pathological results using fluorescence in situ hybridization showed that the COL1A1/PDGFB double-color fusion probe rearrangement was positive, confirming the diagnosis of malignant SFT of the pelvis with high proliferation activity.

## 5. Postoperative course

**Figure 3.**
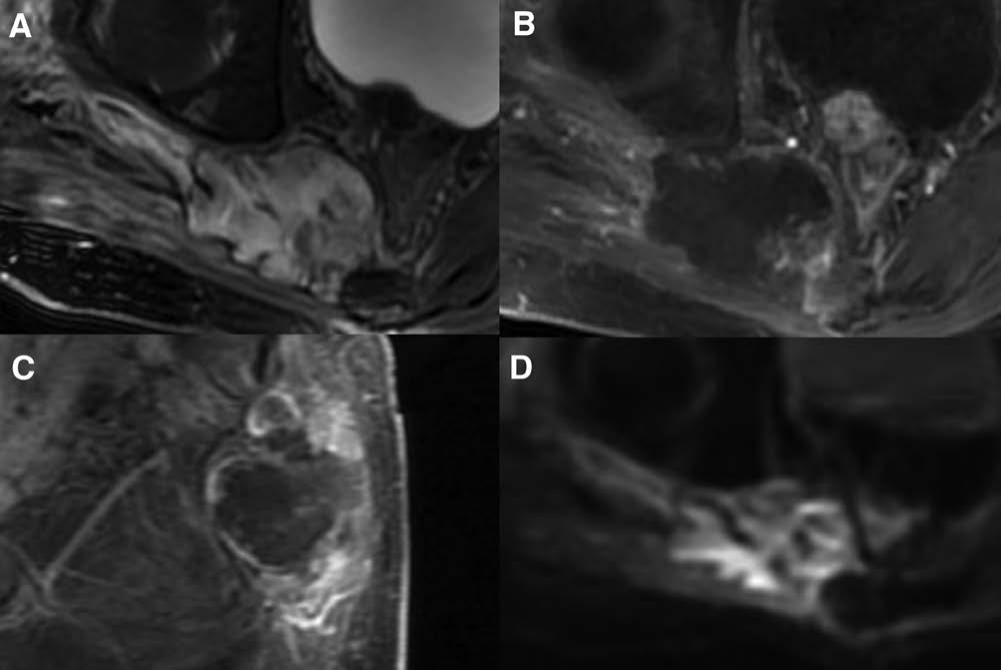
Postoperative MRI revealed infection at the surgical incision site. (A) MRI exhibited infection in the surgical area, which demonstrated mixed intensity on T2-weighted images with high intensity dominated. (B) Contrast-enhanced MR manifested significant enhancement of the lesion margin. (C) Sagittal view of contrast-enhanced MR manifested significant enhancement of the lesion margin. (D) The lesion demonstrated restricted diffusion on diffusion weighted images. MRI = magnetic resonance imaging.

**Figure 4.**
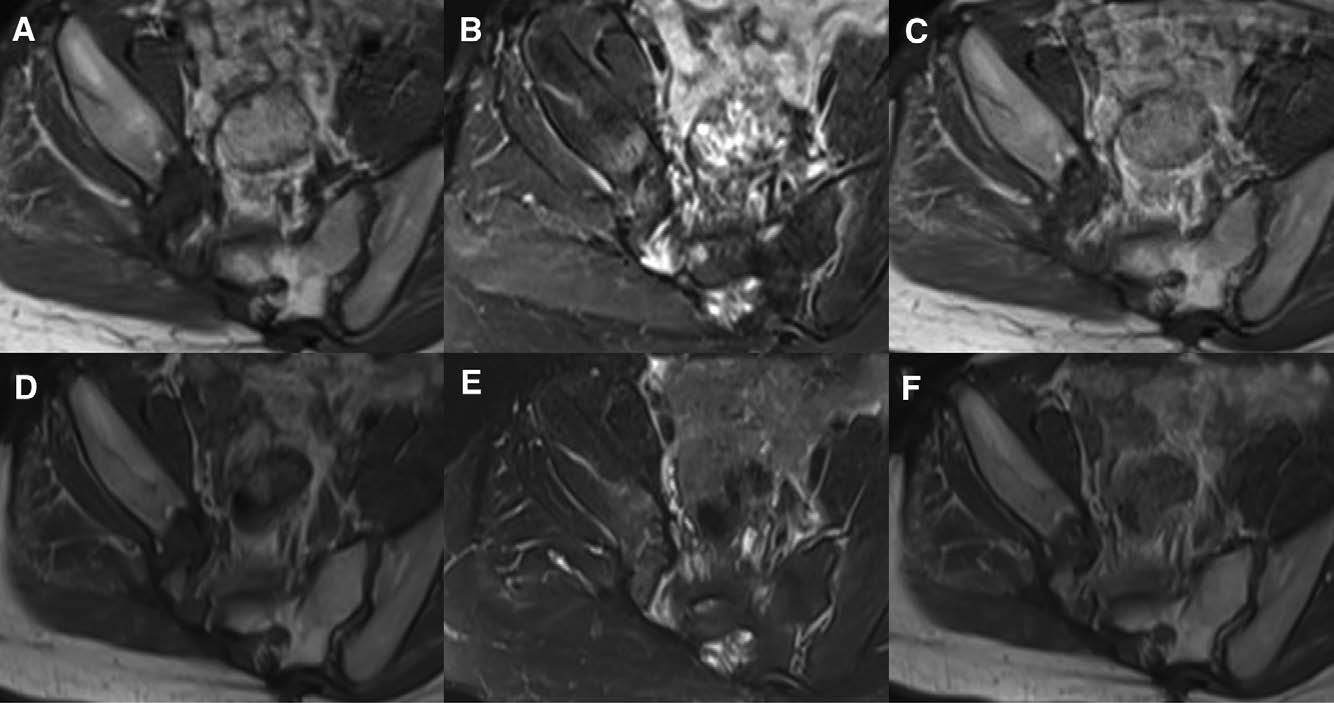
MRI acquired before and after imatinib treatment. (A and B) MRI showed a recurrence of the tumor on the right side of the pelvis, which demonstrated low intensity on T1-weighted images and mixed intensity on T2-weighted images dominated by high intensity. (C) MR contrast-enhanced showed heterogeneous contrast enhancement. (D and E) After treatment with imatinib, the lesion slightly shrank and exhibited low signal on T1-weighted images and slightly high signal on T2-weighted images. (F) MR contrast-enhanced showed heterogeneous contrast enhancement. MRI = magnetic resonance imaging.

Thirteen days after surgery, the patient developed an infection at the surgical incision site (Fig. 3). Subsequently, the patient underwent 5 surgical debridement procedures and 2 thigh skin grafts. Once the anti-infection treatment was initiated, the patient’s inflammatory indexes decreased to normal levels. Follow-up MRI enhancement scanning conducted 9 months after surgery revealed tumor recurrence, with the tumor size decreased from 2.8 × 1.6 × 2.8 cm to 2.1 × 1.1 × 2.7 cm after the patient received imatinib 800 mg per day (400 mg BID) for over a year (Fig. 4).

## 6. Discussion

In this case report, a patient presented with a rare malignant SFT invading sacral canal and foramen, which mimicked a schwannoma on CT. MRI revealed a mixed signal intensity with areas of relatively low T2 signal, which accorded with the pathological finding of collagenous areas. The blood supply was supplied by a branch of the right internal iliac artery, which was consistent with previous literature. However, this case differed from other SFT cases of the pelvis reported before by the presence of recurrence on a 9 months follow-up MRI enhancement. The patient underwent complete surgical excision as the recommended treatment, but recurrence occurred 9 months after surgery. Adjuvant therapy with imatinib 800 mg per day (400 mg BID) was initiated, and subsequent MR contrast-enhanced showed shrinkage of the tumor, indicating that the patient could benefit from this treatment option.

Based on the most recent WHO classification of soft tissue tumors, SFT is now considered an intermediate category tumor, as only a small subset of SFTs exhibit overtly malignant features that could lead to metastasis.^[7]^ SFTs are typically fibroblastic in nature, with a haphazard and storiform arrangement of spindle cells and prominent vascularity, known as the “patternless” pattern.^[8]^ Diagnosis is usually confirmed using immunohistochemistry, which has shown that SFT tumor cells are typically positive for CD34, BCL-2, and CD99, but negative for SMA, desmin, pan-cytokeratin, and S-100 protein.^[9]^ The immunophenotype observed in our case is consistent with past reports.

The classification of soft tissue tumors by the WHO has categorized SFTs into the intermediate (rarely metastasizing) category due to a small subset of SFTs exhibiting malignant characteristics.^[10]^ SFTs are mesenchymal tumors and have been reported to occur in various locations including the lungs, pleura, retroperitoneal space, pelvis, kidney, liver, breast, skin, pancreas, and spinal cord.^[2–4,11]^ The majority of SFTs are benign; however, up to 20% of tumors are malignant. High cellularity, pleomorphism,^[12]^ and high mitotic activity (more than 4 mitoses per 10 HPF) are the pathological criteria associated with malignancy. The size, location, and features of the tumor such as hemorrhage and necrosis can also predict clinical malignancy in extrathoracic SFTs.^[13]^ Malignant SFTs are thought to occur de novo or through dedifferentiation or preexisting histologically benign SFTs. Additionally, the Ki-67 labeling index is usually less than 2% in benign SFTs and more than 6% in malignant SFTs.^[12]^ The current case meets the diagnostic criteria for malignant SFTs.

Typically, extrathoracic SFTs affect both genders equally across all age groups. While most SFTs are asymptomatic, some present with symptoms and signs related to compression on nearby organs.^[14]^ According to prior case reports, approximately 6% of SFTs arise from the pelvis.^[15]^ Malignant SFTs invading the sacral canal and foramen are extremely rare, with only sporadic cases reported.^[6,15–17]^ On CT, SFTs usually appear as heterogeneous masses with 20-30% containing scattered calcifications. In our case, no apparent calcifications were found on CT, consistent with previously reported cases. Most lesions show significant enhancement on CT and MRI due to their rich vascularity. In our case, blood supply was provided by a branch of the right internal iliac artery, as observed in previous literature. However, our case presented as an irregular mass with necrotic areas and invaded the sacral foramen, resembling schwannoma on CT. On MRI, the lesion demonstrated mixed signal intensity with areas of relatively low T2 signal, consistent with the pathological finding of collagenous areas. Notably, these CT or MRI findings are not specific and may be observed in other sacral masses, including but not limited to giant cell tumors of bone, chordoma, and metastatic tumors. This case differed from other reported cases of SFTs in the pelvis due to the presence of recurrence on a 9-month follow-up MRI enhancement.

The recommended treatment for this rare disease entity is complete surgical excision, and the use of adjuvant chemotherapy and radiotherapy for malignant SFTs is controversial due to their rarity, making the benefits difficult to measure.^[16,17]^ To the best of our knowledge, adjuvant chemotherapy has not been previously reported for the treatment of recurrent malignant SFTs of the pelvis. In the present case, the patient underwent adjuvant therapy with imatinib 800 mg per day (400 mg BID) for over a year due to recurrence 9 months after surgery. The follow-up MR contrast-enhanced imaging showed tumor shrinkage, indicating that the patient may have benefited from this treatment option.

## 7. Conclusion

In summary, we presented a rare case of malignant SFT that originated in the sacral foramen of a 15-year-old male patient. The patient underwent surgical excision, and adjuvant therapy with imatinib was administered due to recurrence. Follow-up imaging showed tumor shrinkage, suggesting a positive response to the treatment. Although SFTs are typically benign, malignant cases have been reported, and diagnostic criteria have been established based on pathologic features such as high cellularity, mitotic activity, and pleomorphism. Imaging studies such as CT and MRI are not specific for SFTs, and other pelvic masses should be considered in the differential diagnosis. Complete surgical excision remains the primary treatment option for SFTs, while the use of adjuvant chemotherapy and radiotherapy for malignant cases is controversial. The present case highlights the potential benefits of imatinib as an adjuvant therapy for recurrent malignant SFTs of the pelvic region.

## Author contributions

**Conceptualization:** Panpan Zhou.

**Writing – original draft:** Panpan Zhou, Xiaopei Xu.

## References

[1] Liu B Liu L Li Y. Giant solitary fibrous tumor of the pleura: a case report. Thorac Cancer. 2015;6:368–71. 26273386 10.1111/1759-7714.12175 PMC4448389

[2] Rodríguez AH Martino MD Mazeyra MV. Solitary fibrous tumor of the pancreas. Autops Case Rep. 2021;11:e2021245. 34307213 10.4322/acr.2021.245 PMC8214880

[3] Son S Lee SG Jeong DH. Malignant solitary fibrous tumor of tandem lesions in the skull and spine. J Korean Neurosurg Soc. 2013;54:246–9. 24278657 10.3340/jkns.2013.54.3.246 PMC3836935

[4] Yang LH Dai SD Li QC. Malignant solitary fibrous tumor of breast: a rare case report. Int J Clin Exp Pathol. 2014;7:4461–6. 25120834 PMC4129069

[5] DeVito N Henderson E Han G. Clinical characteristics and outcomes for Solitary Fibrous Tumor (SFT): a single center experience. PLoS One. 2015;10:e0140362. 26469269 10.1371/journal.pone.0140362 PMC4607370

[6] Furuta T Nakai Y Gonoi W. Fat-forming solitary fibrous tumor of the sacrum: a case report and literature review. Radiol Case Rep. 2021;16:1874–7. 34113409 10.1016/j.radcr.2021.04.052 PMC8170013

[7] Fletcher CD. The evolving classification of soft tissue tumours – an update based on the new 2013 WHO classification. Histopathology. 2014;64:2–11. 24164390 10.1111/his.12267

[8] Masuda Y Kurisaki-Arakawa A Hara K. A case of dedifferentiated solitary fibrous tumor of the thoracic cavity. Int J Clin Exp Pathol. 2014;7:386–93. 24427361 PMC3885495

[9] Kawamura S Nakamura T Oya T. Advanced malignant solitary fibrous tumor in pelvis responding to radiation therapy. Pathol Int. 2007;57:213–8. 17316417 10.1111/j.1440-1827.2007.02083.x

[10] Vossough A Torigian DA Zhang PJ. Extrathoracic solitary fibrous tumor of the pelvic peritoneum with central malignant degeneration on CT and MRI. J Magn Reson Imaging. 2010;22:684–6. 10.1002/jmri.20433 16215968

[11] Hsieh TY ChangChien YC Chen WH. De novo malignant solitary fibrous tumor of the kidney. Diagn Pathol. 2011;6:96. 21970525 10.1186/1746-1596-6-96 PMC3195699

[12] England DM Hochholzer L Mccarthy MJ. Localized benign and malignant fibrous tumors of the pleura. A clinicopathologic review of 223 cases. Am J Surg Pathol. 1989;13:640–58. 2665534 10.1097/00000478-198908000-00003

[13] Guillou L Fletcher JA Fletcher CDM. Extrapleural solitary fibrous tumor and hemangiopericytoma. In: Fletcher CDM Unni KK Mertens F, eds. Pathology and Genetics of Tumours of Soft Tissue and Bone. Lyon: IARC Press; 2002:86–90.

[14] Gold JS Antonescu CR Hajdu C. Clinicopathologic correlates of solitary fibrous tumors. Cancer. 2002;94:1057–68. 11920476

[15] Gao C Zhang Y Jing M. Postoperative radiotherapy for the treatment of solitary fibrous tumor with malignant transformation of the pelvic: a rare case report with literature review. Medicine (Baltim). 2016;95:e2433. 10.1097/MD.0000000000002433 PMC4718252 26765426

[16] Boe J Chimpiri AR Liu C. Solitary fibrous tumor originating in the pelvis: a case report. J Radiol Case Rep. 2010;4:21–8. 10.3941/jrcr.v4i7.430 PMC3303364 22470743

[17] Pata F Orsini V Lucisano AM. Solitary fibrous tumor of the pelvis: an uncommon soft-tissue tumor. A case report. Ann Ital Chir. 2010;81:457–60. 21456483

